# Supplementary material for: Sequential regression and simulation: a method for estimating causal effects from heterogeneous clinical trials without a common control group
Source: BMC Med Res Methodol. 2023 Oct 3;23:218. doi: 10.1186/s12874-023-02020-5 (PMC10546672; doi:10.1186/s12874-023-02020-5)
Supplement: Supplementary file 1 — Additional file 1: Supplementary methods. [file 12874_2023_2020_MOESM1_ESM.pdf]

SUPPLEMENTARY METHODS:

QUALITY CONTROL, HARMONIZATION, MISSING DATA

We performed extensive quality control evaluations of the included trials and data (Figure 1a). This included confirming our ability to reproduce published statistics on the trial cohorts at baseline as well as the study primary endpoint (Supplementary Figure 3, Additional File 2). We were able to exactly reproduce most of the study results. Where discrepancies occurred, they were generally minor and fell within a 10% error bound. We reported major discrepancies to the study sponsor as per agreement. We attempted to completely eliminate all discrepancies, but this was not possible due a variety of factors, including lack of access to the original analytic code or the complete analytic dataset, and inability to contact the original analysts.

We completed an assessment of data availability for all study variables (Supplementary Figure 4, Additional File 2). Target variables included demographic features, CDAI at baseline and week eight, baseline inflammatory biomarkers, concomitant steroid and immunomodulator use, history of treatment with tumor necrosis factor-alpha inhibitors (TNFis), and other disease-related features. We identified nine variables that were universally available across all trials and thus could be used for downstream modeling: Age, Sex, BMI, baseline CDAI, c-reactive protein (CRP), history of TNFi use, oral steroid use, immunomodulator use, and ileal involvement.

Only 3% of the participants had at least one missing covariate at baseline. Continuous variables were addressed by median imputation, and participants with missing categorical variables were dropped from the dataset (N=86). 11% of the participants had a missing value for the outcome at week eight. To handle this, we used last-observation-carried-forward to impute these values, typically using measurements from week six and four. This is the typical practice for the analysis of these trials in regulatory submissions and was the prespecified approach in the protocols for all included trials. The variable corresponding to a history of TNFi use was available in all recent trials that occurred after the approval of the very first TNFi medication. Older trials of the first TNFis commonly excluded patients who had a history of exposure to other drugs from this class but did not include this feature as an actual variable in the data set. In these cases, we deterministically imputed this variable corresponding to no prior use.

Other variables of a priori importance could not be included in this study. Ethnicity was not collected in most trials. Race was missing in some trials, but when it was captured, it reflected significant imbalance (88% of participants were white). Other disease specific variables such as disease behavior and duration were also not uniformly captured across studies and thus could not be included in this meta-analysis.

STATISTICAL COMPUTING

Programming was performed in the R language, using the packages *dplyr*[1], *lme4*[2], *lmerTest*[3], *data.table*[4], *ggplot2*[5], *ggpubr*[6], *sjstats*[7], *patchwork*[8], and *gridExtra*[9]. The analytical code was independently reviewed by a second member of the team.

MODEL FOR ESTIMATING THE PLACEBO EFFECT

We fit a linear mixed effect model to predict the placebo effect on each patient's CDAI reduction at week 8. The model was trained on the placebo arms of the six placebo-controlled trials. We denote them as trial 1 to trial 6 to simplify the notation. The CDAI reduction of patient j from trial i in the placebo arm at week 8 is denoted as $y_{ij}^{placebo}$ and assumed to be related to the nine predictors $D_{ij,1}, \dots, D_{ij,9}$, the centered study year T_i, and the trial-specific random effect S_i as in the following equation:

$$y_{ij}^{placebo} = \beta_0 + \beta_1 D_{ij,1} + \beta_2 D_{ij,2} + \cdots + \beta_9 D_{ij,9} + \gamma_i T_i + S_i + \epsilon_{ij},$$
$$i = 1, \dots, 6;\ j = 1, \dots, n_i \qquad (1)$$

Where $\epsilon_{ij} \sim N(0, \sigma_\epsilon^2)$, $S_i \sim N(0, \sigma_{s1}^2)$, and n_i is the sample size of each trial, respectively.

MODEL FOR ESTIMATING ADALIMUMAB DRUG-ATTRIBUTABLE EFFECT

After fitting the placebo-effect model, we used the coefficients of model (1) to predict the placebo-attributable component of the observed outcomes of the participants from three study cohorts assigned to receive adalimumab at the FDA-approved dose for treatment induction. We name them as trial 7 to trial 9 to simplify the notations. Denoting the observed CDAI reduction at week 8 of patient j from trial i as y_{ij} and the predicted placebo-attributable component as $y_{ij}^{placebo}$, we assume the difference $\epsilon_{ij} = y_{ij} - y_{ij}^{placebo}$ reflects the adalimumab drug-attributable effect and is related to the same nine predictors and trial-specific random effect of each adalimumab trial as in the equation below:

$$\epsilon_{ij} = \theta_0 + \theta_1 D_{ij,1} + \theta_2 D_{ij,2} + \ldots + \theta_9 D_{ij,9} + S_i + \xi_{ij}$$
$$i = 7,8,9;\ j = 1, \ldots, n_i \qquad (2)$$

Where $\epsilon_{ij} \sim N(0, \sigma_\xi^2)$, $S_i \sim N(0, \sigma_{s2}^2)$, and n_i is the sample size of each trial, respectively.

MODEL FOR EXTERNAL VALIDATION

To emulate SEAVUE, we identified all placebo-arm participants from the three ustekinumab-related trials who were biologic-naive as the simulated adalimumab cohort. The observed CDAI reduction of the participants at week 8 are denoted as $y_k^{placebo}$, where $k = 1, \ldots, 135$. We then use the coefficients of model (2) to predict the adalimumab drug-attributable effect of the simulated cohort and denote it as $\hat{e}_k$. The CDAI reduction at week 8 of each simulated adalimumab participant is calculated by $\hat{y}_k = y_k^{placebo} + \hat{e}_k$. The number of remission N_{rem} is calculated by the count of $BaselineCDAI_k - \hat{y}_k \leq 150$. The remission rate is calculated by $N_{rem}/135$.

REFERENCES:

1 Wickham H, François R, Henry L, Müller K. dplyr: A Grammar of Data Manipulation. Published online 2022. https://dplyr.tidyverse.org

2 Bates D, Mächler M, Bolker B, Walker S. Fitting Linear Mixed-Effects Models Using lme4. *J Stat Softw*. 2015;67:1-48. doi:10.18637/jss.v067.i01

3 Kuznetsova A, Brockhoff PB, Christensen RHB. lmerTest Package: Tests in Linear Mixed Effects Models. *J Stat Softw*. 2017;82:1-26. doi:10.18637/jss.v082.i13

4 Dowle M, Srinivasan A, Gorecki J, et al. data.table: Extension of "data.frame." Published online September 27, 2021. Accessed August 29, 2022. https://CRAN.R-project.org/package=data.table

5 Wickham H. *Ggplot2: Elegant Graphics for Data Analysis*. Springer; 2016.

6 Kassambara A. ggpubr: “ggplot2” Based Publication Ready Plots. Published online June 27, 2020. Accessed August 29, 2022. https://CRAN.R-project.org/package=ggpubr

7 Lüdecke D. sjstats: Collection of Convenient Functions for Common Statistical Computations. Published online January 9, 2021. Accessed August 29, 2022. https://CRAN.R-project.org/package=sjstats

8 Pedersen TL. patchwork: The Composer of Plots. Published online August 19, 2022. Accessed August 29, 2022. https://CRAN.R-project.org/package=patchwork

9 Auguie B, Antonov A. gridExtra: Miscellaneous Functions for “Grid” Graphics. Published online September 9, 2017. Accessed August 29, 2022. https://CRAN.R-project.org/package=gridExtra
