# Supplementary material for: Sequential regression and simulation: a method for estimating causal effects from heterogeneous clinical trials without a common control group
Source: BMC Med Res Methodol. 2023 Oct 3;23:218. doi: 10.1186/s12874-023-02020-5 (PMC10546672; doi:10.1186/s12874-023-02020-5)
Supplement: Supplementary file 2 — Additional file 2: Figure S1. PRISMA-IPD flow diagram. Figure S2. Risk of bias. Figure S3. Reproducibility of published data. Figure S4. Leave-one-trial-out analysis. Figure S5. Checking model assumptions. Table S1. Major inclusion/exclusion criteria of included studies. Table S2. Model selection. [file 12874_2023_2020_MOESM2_ESM.pdf]

Supplementary Figure 1: PRISMA-IPD flow diagram

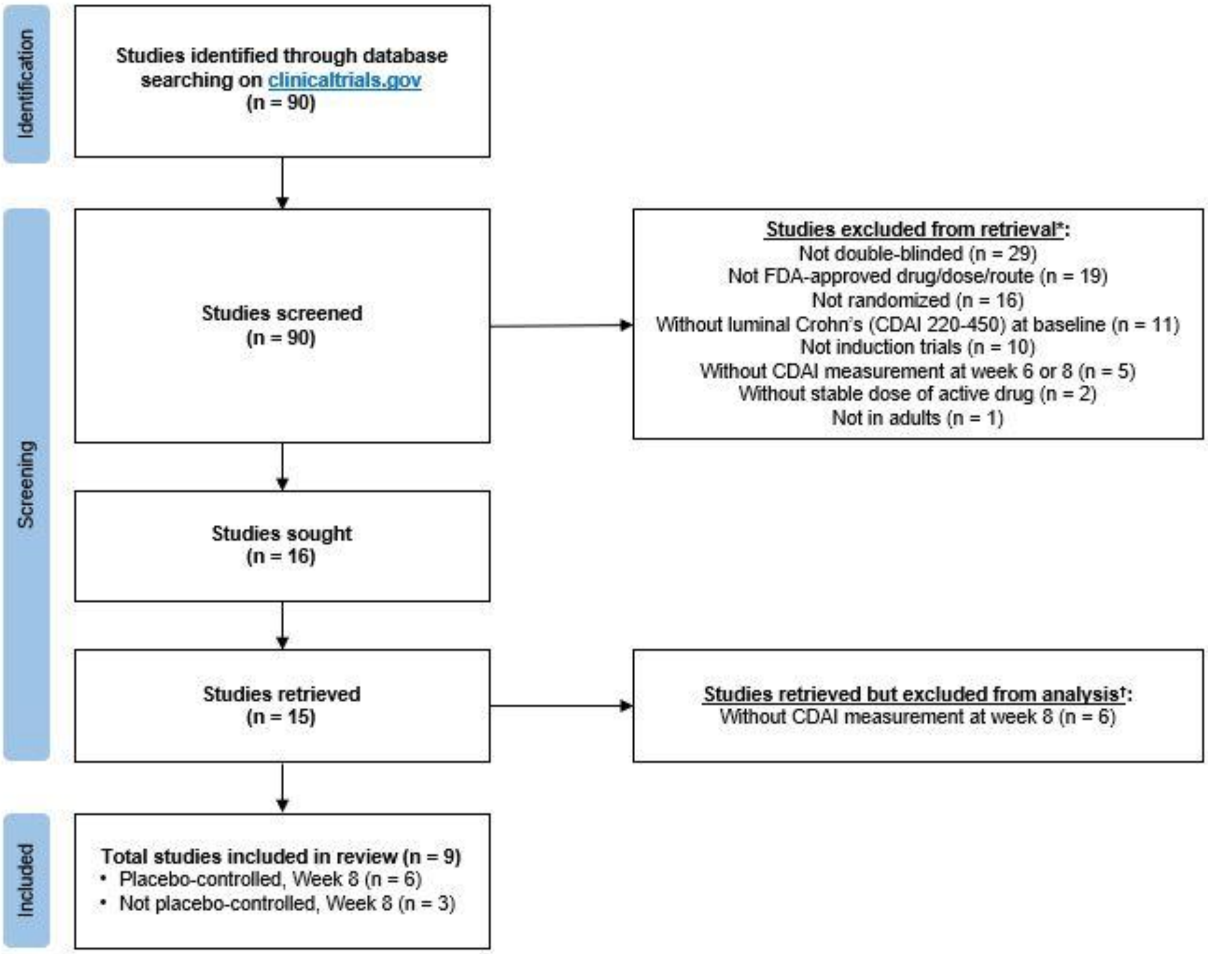

Flow diagram illustrating selection of studies.

*Some studies met more than one criterion.
†All 15 studies were retrieved and consolidated on the Vivli platform; however, only 9 studies were used for analysis as these studies captured CDAI measurement at week 8 and could be compared with the SEAVUE study.

Supplementary Figure 2: Risk of bias

		Randomization Process	Deviations from intended interventions	Missing outcome data	Measurement of the outcome	Selection of the reported result	Outcome
NCT01369329	UNITI1	+	+	+	+	+	+
NCT01369342	UNITI2	+	+	+	+	+	+
NCT00771667	CERTIFI	+	+	+	+	+	+
NCT00783692	GEMINI2	+	+	+	+	+	+
NCT01224171	GEMINI3	+	+	+	+	+	+
NCT00032786	ENACT	+	+	+	+	+	+
NCT00078611	ENCORE	+	+	+	+	+	+
NCT00552058		+	+	+	+	+	+
NCT00291668		+	+	+	+	+	+
NCT00152490	PRECISE1	!	+	+	!	+	+
NCT00152425	PRECISE2	-	!	+	+	+	-
NCT00207662	ACCENT	-	!	+	+	+	-
NCT00094458	SONIC	+	+	+	+	+	+
NCT00348283	EXTEND	-	!	+	+	+	+
NCT00055523	CLASSIC1	+	+	+	+	+	+
NCT00055523	CLASSIC2	-	!	+	!	!	-
NCT02499783		+	+	+	+	+	+

Cochrane's risk-of-bias tool for randomized trials version 2 (ROB2). Green, yellow, and red indicate low, moderate, and high risk of bias respectively.

Supplementary Figure 3: Reproducibility of published data

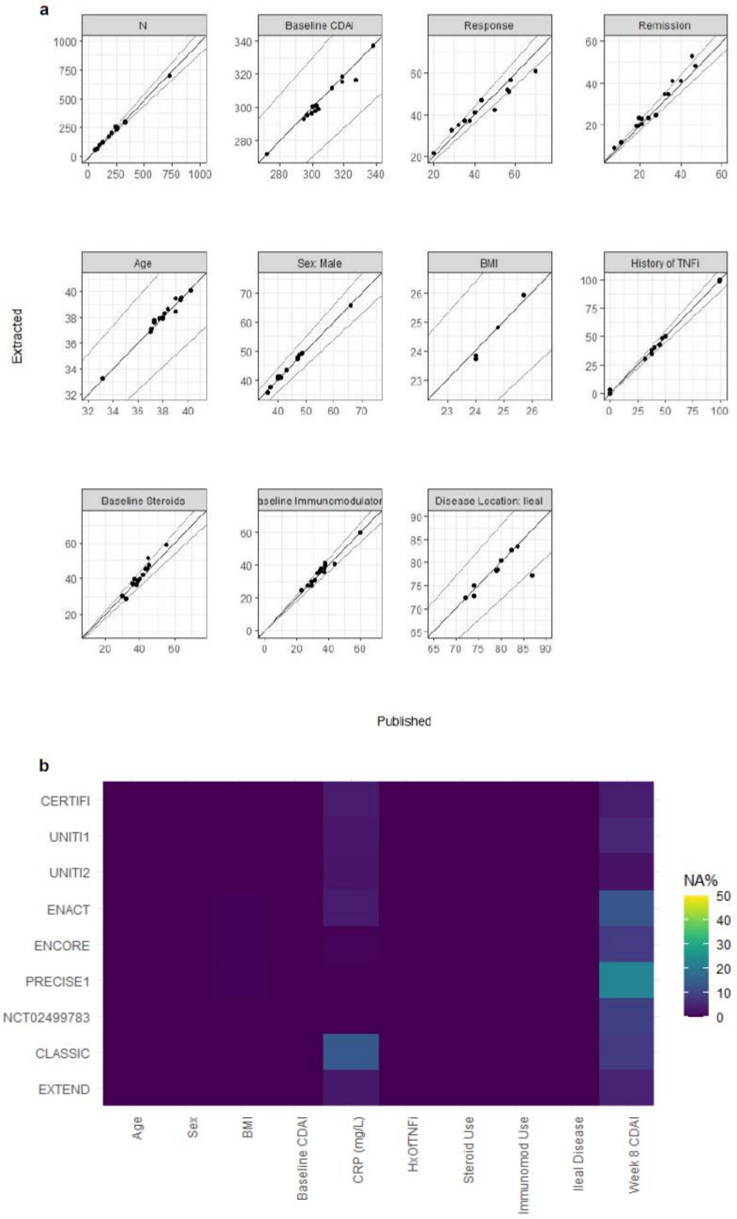

a, Plots of aggregated data versus published data for baseline covariates and outcome variables as a measure of quality control. Each dot represents the mean variable estimate for a given study treatment group (placebo, active). Data were not displayed if the study did not report the variable mean in its original article. Upper and lower lines in plots correspond to ±10% error bounds. **b**, Percentage of missing covariates by study. Approximately 0.2% of BMI values, 2% of CRP values, and 11% of week 8 CDAI values were missing after data harmonization and required imputation. Median imputation by study was used to impute missing BMI and CRP values. Last observation carried forward (LOCF) was used to impute missing week 8 CDAI values; CDAI observations from week 6, week 4, week 3, or week 2 were candidates for LOCF.

Supplementary Figure 4: Leave-one-trial-out analysis

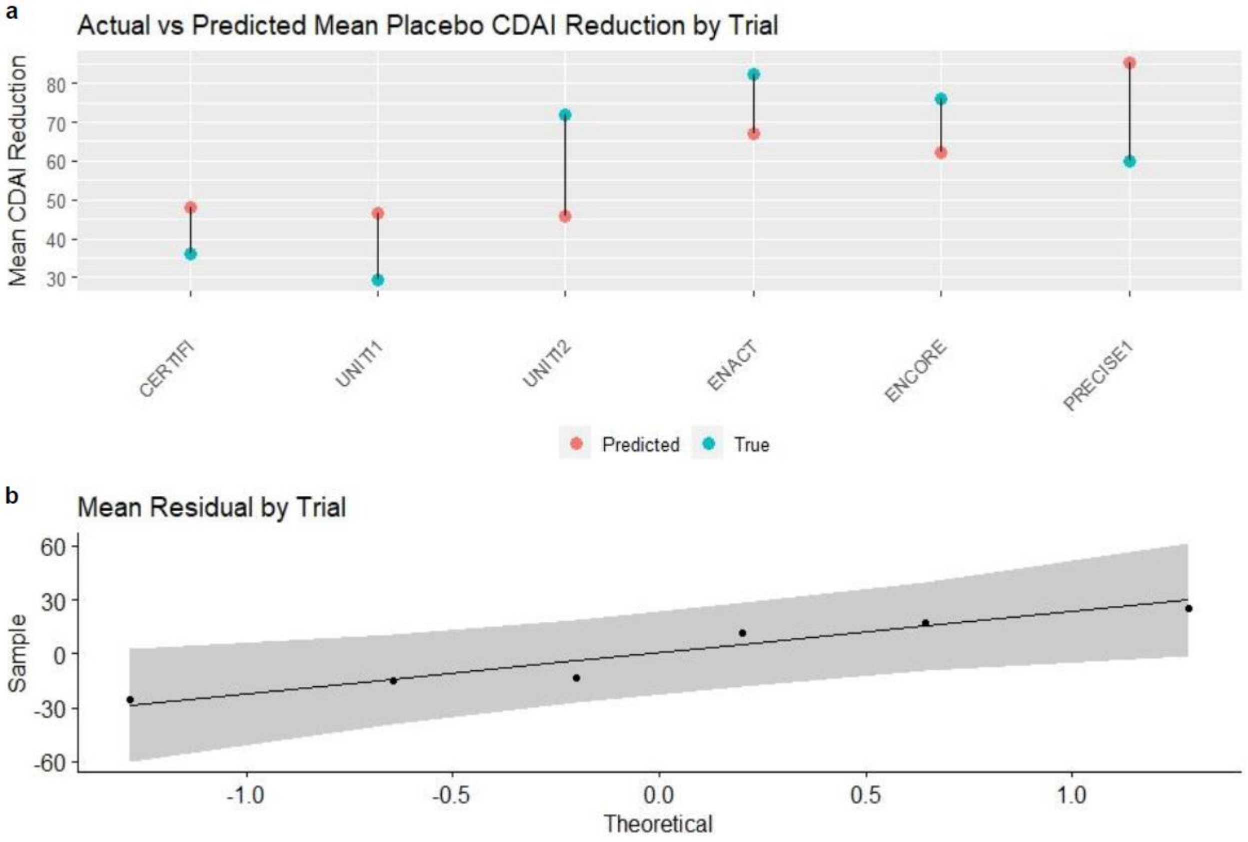

a, A leave-one-trial-out analysis, where blue and red dots represent the true and predicted mean CDAI reduction respectively. **b**, Q-Q plot of the model residuals (difference in true and predicted mean CDAI reduction values per study) to assess residual normality (p=0.4 by the Shapiro-Wilk test).

Supplementary Figure 5: Checking model assumptions

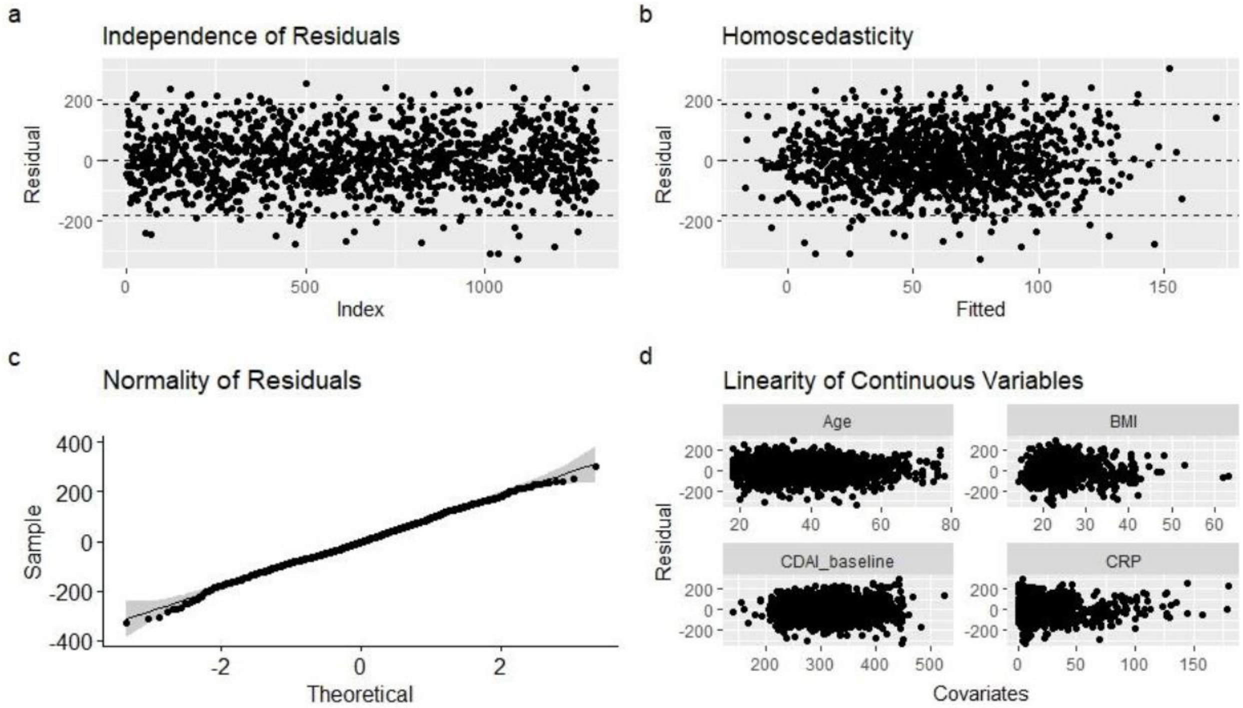

Plotting the placebo-attributable model residuals to visually check linear regression model assumptions. **a**, Plot of the model residuals versus index to assess residual independence. Dotted lines represent $\pm 2\boldsymbol{\sigma}$. **b**, Plot of the model residuals versus the fitted values to assess residual homoscedasticity. **c**, Q-Q plot of the model residuals to assess residual normality. **d**, Plot of the model residuals versus each continuous covariate to assess linearity between covariates and the outcome variable.

Supplementary Table 1: Major inclusion/exclusion criteria of included studies

Trial	Age ≥ 18	Baseline CDAI 220-450	Ileal and/or colonic involvement	Disease activity by biochemical/ imaging/ endoscopy	TNFi intolerance/ failure	TNFi naive*	Stable concomitant medications	No symptomatic stricture	No abscess	No recent surgery	No stoma or ostomy
PRECISE1	✓	✓	NA	NA	✗	✓	✓	✓	✓	NA	✓
ENACT	✓	✓	✓	✓	✗	✗	✓	✓	✓	NA	✓
ENCORE	✓	✓	NA	✓	✗	✗	✓	✓	✓	✗	✓
CERTIFI	✓	✓	✓	✗	✓	✗	✓	✓	✓	✓	✓
UNITI1	✓	✓	✓	✗	✓	✗	✓	✓	✓	✓	✓
UNITI2	✓	✓	✓	✓	✗	✓	✓	✓	✓	✓	✓
CLASSIC	✓	✓	NA	NA	✗	✓	✓	✓	NA	✓	✓
EXTEND	✓	✓	✓	✓	✗	✗	✓	NA	NA	NA	NA
NCT02499783	✓	✓	✗	✓	✗	✓	✓	✓	✗	✓	✓

If the trial protocol was not publicly available, and if the corresponding manuscript or clinical study report was silent on a given criteria, the field was annotated as NA. If the trial protocol was available and if it was clear that a given criterion was not applied for cohort selection, the field was annotated with an X. In many trials (e.g., ENACT, ENCORE), a history of TNF-naive or intolerance/failure was not a requirement and was captured as a participant-specific covariate for regression-based control. In other scenarios, the trial-specific covariate implicitly applied to all trial participants (e.g., TNF-naive status in PRECISE1). Trials were generally consistent on the target patients of study in terms of inclusion and exclusion criteria. To address the possibility of residual heterogeneity due to the lack of perfectly consistent eligibility criteria or unmeasured covariates, a trial-specific random effect was included in the final regression models.

*Either absence of exposure or absence of prior intolerance or inadequate response.

Supplementary Table 2: Model selection

Prediction Model	Package	Mean 5-Fold CV RMSE
Linear Regression	lm	93.19
Linear Mixed Effect Model	lme4	93.19
Linear LASSO Regression	glmnet	93.14
Random Forest	randomForest	92.85
XGBoost (DART)	xgboost	96.20
Stacked Ensemble	MLJAR	90.81

Mean 5-fold cross-validation (CV) root mean squared error (RMSE) scores of various predictive models. All models were tested against the same stratified 5-fold datasets to ensure results were comparable. RMSE scores reflect the model's ability to predict CDAI reduction at week 8 due to a placebo treatment given baseline covariates (age, sex, BMI, etc.). The stacked ensemble model was built from 9 default base models using the mljar AutoML package.
